# Public Discourse Toward Older Drivers in Japan Using Social Media Data From 2010 to 2022: Longitudinal Analysis

**DOI:** 10.2196/69321

**Published:** 2025-06-16

**Authors:** Akito Nakanishi, Masao Ichikawa, Yukie Sano

**Affiliations:** 1 Graduate School of Science and Technology, University of Tsukuba, Ibaraki, Japan; 2 Institute of Medicine, University of Tsukuba, Ibaraki, Japan; 3 Institute of Systems and Information Engineering, University of Tsukuba, 1-1-1 Tennodai, Tsukuba, Ibaraki, 3058573, Japan, 81 29-853-5552

**Keywords:** older driver, older people, ageism, social media, Twitter, sentiment analysis, topic modeling

## Abstract

**Background:**

As the global population ages, concerns about older drivers are intensifying. Although older drivers are not inherently more dangerous than other age groups, traditional surveys in Japan reveal persistent negative sentiments toward them. This discrepancy suggests the importance of analyzing discourse on social media, where public perceptions and societal attitudes toward older drivers are actively shaped.

**Objective:**

This study aimed to quantify long-term public discourse on older drivers in Japan through Twitter (subsequently rebranded X), a leading social media platform. The specific objectives were to (1) examine the sentiments toward older drivers in tweets, (2) identify the textual contents and topics discussed in the tweets, and (3) analyze how sentiments correlate with various variables.

**Methods:**

We collected Japanese tweets related to older drivers from 2010 to 2022. Each quarter, we (1) applied to the Japanese version of the Linguistic Inquiry and Word Count dictionary for sentiment analysis, (2) employed 2-layer nonnegative matrix factorization for dynamic topic modeling, and (3) applied correlation analyses to explore the relationships of sentiments with crash rates, data counts, and topics.

**Results:**

We obtained 2,625,807 tweets from 1,052,976 unique users discussing older drivers. The number of tweets has steadily increased, with significant peaks in 2016, 2019, and 2021, coinciding with high-profile traffic crashes. Sentiment analysis revealed a predominance of negative emotions (n=383,520, 62.42%), anger (n=106,767, 17.38%), anxiety (n=114,234, 18.59%), and risk (n=357,311, 58.15%). Topic modeling identified 29 dynamic topics, including those related to driving licenses, crash events, self-driving technology, and traffic safety. The crash events topic, which increased by 0.28% per year, showed a strong correlation with negative emotion (*r*=0.76, *P*<.001) and risk (*r*=0.72, *P*<.001).

**Conclusions:**

This 13-year study quantified public discourse on older drivers using Twitter data, revealing a paradoxical increase in negative sentiment and perceived risk, despite a decline in the actual crash rate among older drivers. These findings underscore the importance of reconsidering licensing policies, promoting self-driving systems, and fostering a more balanced understanding to mitigate undue prejudice and support continued safe mobility for older adults.

## Introduction

As the global population ages, concerns about older drivers are increasing. The share of the global population aged 70 years or older is projected to rise from 6.4% in 2023 to 11.7% in 2050 [1]. Japan is one of the most rapidly aging societies, with 23.6% of its population being 70 years or older in 2023. Concurrently, the proportion of driver’s license holders among older Japanese people is also increasing, reaching 16.6% for those aged 70 years or older in 2023 [2]. In response to their decline in physical and cognitive functions, as well as tragic traffic crashes involving them, the National Police Agency of Japan has incrementally tightened licensing policies for them in 1998, 2002, 2009, 2017, and 2022 [3].

Research on older drivers’ traffic safety frequently examines their risk of traffic crashes, neurological and physical impairments, and crash causes. A global meta-review [4] finds that older drivers have a higher risk of crashes and injuries even when controlling for travel time or distance, partly because they experience declines in vision, cognitive performance, reaction time, and physical ability, despite their strong adherence to traffic laws. This makes older drivers a higher-risk group in traffic than younger drivers, who are more likely to be involved in crashes due to law violations or careless behavior. In addition to traffic crashes, studies have also discussed the effects of driving cessation, such as increased depressive symptoms [5,6] and reduced quality of life [7]. In Japan, research on older drivers is a relatively new field [8] and covers a range of topics, including dangerous driving associated with dementia [9], qualitative surveys of older drivers and their families [8,10,11], and discussions on licensing policies [12,13].

**Figure 1.**
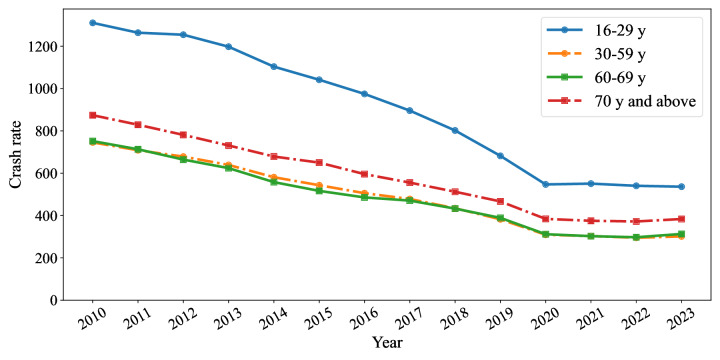
Traffic crash rates per 100,000 licensed drivers by year, categorized by age group (16‐29 y, 30‐59 y, 60‐69 y, and 70 y and above).

Although many studies highlight the risks associated with older drivers, they are not significantly more dangerous than other age groups in Japan when considering both their at-fault crash rate and the harm they cause to others. As shown in Figure 1, the at-fault crash rate (per 100,000 licensed drivers) for car and motorcycle drivers aged 70 years or older was 384 in 2023, which was higher than for those in middle age groups (30‐59 y: 301 and 60‐69 y: 313), but lower than for those in younger age groups (16‐19 y: 1025 and 20‐29 y: 497) [14]. Furthermore, the rate among those aged 70 years or older substantially decreased in the past decades from 874 in 2010 to 384 in 2023. It is also noted that their at-fault fatal crashes tend to result in fewer fatalities for occupants of other vehicles compared with fatal crashes caused by drivers in other age groups [15]. These statistics demonstrate that the risk of traffic crashes and resultant injuries imposed by older drivers is not as high as perceived, as driving failure frequencies do not significantly differ between age groups [4].

Despite the decreasing crash rate, negative sentiments toward older drivers are still persistent in Japan. According to a survey by the National Police Agency, 85% (n=1698) of respondents (balanced by age and sex) perceive older drivers as dangerous, and 80% (n=1596) believe that licensing for older drivers should be revised [16]. Such sentiments are potentially influenced by media reports on older drivers and their crashes as well as stereotypes about older people. Recent studies suggested a negative tone toward older drivers in news reports [17], an increase in newspaper articles about them [18], and underreported of their crashes killing themselves [19]. However, discourse regarding older drivers in social media has not been explored despite the widespread use of various social media platforms.

Today, social media plays various roles in formal and informal communication at individual, organizational, and societal levels. The number of social media users escalates globally [20], with nearly 50% of Japan’s population using Twitter (now X), a leading text-based social media platform [21]. Social media not only facilitates access to a broad range of information but also serves as a platform for public discussion, allowing individuals to express their views, opinions, and sentiments. As the reliance on online health information also intensifies [22], research into health-related topics on Twitter has expanded to include discussions on food security [23], type 1 diabetes [24], and various aspects of COVID-19, such as mask-wearing [25], vaccines [26-28], and the pandemic [29]. Consequently, applying text mining techniques, frequently employed to quantify the textual content and sentiment of tweets in these fields, offers valuable insights to facilitate a direct interpretation of societal discussions around health-related issues.

Given social media’s impacts on public opinions and policy-making, gaining insights on older drivers and their crashes from social media—beyond conventional surveys [17,30]—is crucial for strategic planning of traffic safety. In this study, we aimed to (1) examine the sentiments toward older drivers in tweets, (2) identify the textual contents and topics discussed in the tweets, and (3) analyze how sentiments correlate with various variables. To achieve these goals, we conducted sentiment analysis, topic modeling, and correlation analysis, using over 2.6 million tweets on older drivers posted in the past 13 years.

## Methods

### Study Workflow

**Figure 2.**
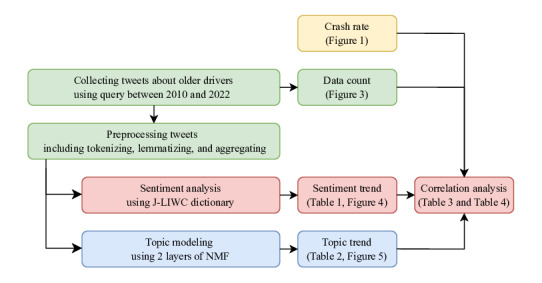
Diagram of the study workflow. Tweets are collected and preprocessed, followed by sentiment analysis, topic modeling, and correlation analysis. Each figure and table number corresponds to the main text. J-LIWC: Japanese version of the Linguistic Inquiry and Word Count Dictionary; NMF: nonnegative matrix factorization.

Figure 2 illustrates the workflow of this study, covering data collection and processing, sentiment analysis, topic modeling, and correlation analysis.

### Data Collection and Processing

We collected tweets about older drivers posted between January 1, 2010 and December 31, 2022, using the Twitter application programming interface v2 [31] with Japanese keywords “(高齢 OR 老人) AND (運転 OR ドライバー)”, or literally, “(old age OR older people) AND (driving OR driver).” To ensure the relevance of collected tweets, we examined the contents in a random sample of 1000 tweets and determined that 967 tweets pertained to older drivers or their transportation issues.

As part of text preprocessing, we removed symbols, emojis [32], URLs, and hashtags (#...), and normalized characters by converting them to lowercase and half-width forms. Tweets were then tokenized into words using MeCab [33] with the mecab-ipadic dictionary, with stopwords removed (Multimedia Appendix 1) and verbs and adjectives lemmatized. In addition, we consolidated tweets from the same user within the same quarter by merging their tokenized word lists into single documents. This approach reduces individual biases, enables dynamic trend analysis by quarter, and improves topic modeling accuracy by processing aggregated and extended textual content [34]. After pooling tweets by user and quarter, we removed words with an occurrence rate of less than 0.1% per quarter, resulting in 9287 unique words. Hereafter, we refer to the collection of quarterly tweets aggregated by each user as a “document.”

### Sentiment Analysis

To clarify the Twitter users’ feelings toward older drivers, we first conducted sentiment analysis using the Japanese version of the Linguistic Inquiry and Word Count Dictionary (J-LIWC) [35]. Sentiment analysis quantifies the sentiment expressed in documents, using techniques ranging from word-based and context-based methods to deep-learning approaches. We adopted a word-based method for its interpretability and consistency with topic modeling, specifically using J-LIWC, which is psychologically validated and reliable in both English and Japanese.

The J-LIWC includes 69 categories in 4 broad categories—“psychological processes” (37 categories), “linguistic processes” (14 categories), “punctuation” (12 categories), and “other grammar” (6 categories). For this study, we used the “psychological processes” of the J-LIWC, aligning with our research objectives. The “psychological processes” consists of 10 subcategories, namely “affective processes,” “negative emotions,” “social processes,” “cognitive processes,” “perceptual processes,” “biological processes,” “drives,” “relativity,” “personal concerns,” and “informal language,” and under these subcategories, there are several further subcategories, as illustrated in Table 1. Throughout this paper, we refer to the further subcategories of the J-LIWC as sentiments.

**Table 1.** Proportion of documents containing words corresponding to 10 sentiments in the Japanese version of the Linguistic Inquiry and Word Count Dictionary’s “affective processes,” “negative emotions,” and “drives” within 614,429 documents discussing older drivers posted from 2010 to 2022. In addition to proportions, results of linear regression (the formula $Y_{t}=\beta_{0}+\beta_{1}X_{t}+\epsilon_{t}$) are represented by $\beta_{0},\beta_{1}$, *R*^2^, and *P* values.

Subcategory and sentiment			Tweets, n (%)	$\beta_{0}$	$\beta_{1}$	*R* ^2^	*P* value
Affective processes							
	Positive emotions		217,300 (35.37)	31.0	0.11	0.154	.004
	Negative emotions		383,520 (62.42)	46.1	0.36	0.462	<.001
Negative emotions							
	Anxiety		114,234 (18.59)	14.0	0.12	0.395	<.001
	Anger		106,767 (17.38)	11.0	0.17	0.686	<.001
	Sadness		23,609 (3.84)	1.70	0.05	0.230	<.001
Drives							
	Affiliation		105,984 (17.25)	15.6	0.04	0.049	.120
	Achievement		179,969 (29.29)	21.7	0.18	0.416	<.001
	Power		213,716 (34.78)	28.1	0.15	0.362	<.001
	Reward		105,067 (17.10)	13.1	0.10	0.412	<.001
	Risk		357,311 (58.15)	42.8	0.33	0.384	<.001

### Topic Modeling

Next, we employed 2 layers of nonnegative matrix factorization (NMF) [36], a form of dynamic topic modeling. Topic modeling aims to extract latent textual data and generate topic distributions categorized as “static” (ignoring time) and “dynamic” (incorporating temporal variation) [37,38]. While many previous studies use latent Dirichlet allocation [39], it does not account for temporal trends, thus unsuitable for longitudinal studies. Several models have been developed to capture topic dynamics over time, such as dynamic topic model (DTM) [40], which is an extended version of latent Dirichlet allocation, 2 layers of NMF [35], and BERTopic [41]. DTM and 2 layers of NMF rely on a bag-of-words approach, whereas BERTopic uses word embeddings. This study opted for 2 layers of NMF as it provides a richer set of top words, higher coherence scores, faster performance than DTM, and, compared with BERTopic, offers objective evaluation metrics, enables topic distribution generation for each document, and eliminates the need for labor-intensive testing [36,42].

Two layers of NMF is a method for extracting dynamic topic distributions by applying a 2-stage NMF, an unsupervised dimensionality reduction technique that decomposes data into nonnegative factors. In the first stage, document-term matrix $A_{t}$ for each time unit (quarter) *t* undergoes NMF, producing a “window topic,” which represents topic distributions within each time unit. In the second stage, all “window topic”–term matrix B (a combination of all $A_{t}$) are further decomposed via NMF, resulting in a “dynamic topic,” which incorporates temporal changes.

Following the previous study [36], we determined the optimal number of dynamic topics by testing a range between 25 and 90, selecting the one with the highest Topic Coherence using Word2Vec score [43]. TC-W2V score is defined as the mean pairwise cosine similarity between term vectors embedded using the word2vec model [44]. In this study, we employed the Japanese Social Media Corpus [45] as a large-scale word2vec model, trained on approximately 2 million Japanese words from social media and web sources (Multimedia Appendix 1).

### Statistical Tests

In each section, we employ simple linear regression to analyze temporal trends in data counts, sentiments, and topics. The simple linear regression model is expressed as follows:


$$Y_{t}=\;\beta_{0}+\beta_{1}X_{t}+\epsilon_{t}$$

where $Y_{t}$ represents the dependent variable (eg, tweet count at quarter *t*) and $X_{t}$ denotes the independent variable (eg, quarter *t*). The coefficient $\beta_{1}$ indicates the rate of change, while the intercept $\beta_{0}$ represents the baseline value when $X_{t}$ is zero. The term $\epsilon_{t}$ accounts for random fluctuations not explained by the model. For this analysis, we use the actual data count, the proportion of documents containing a given sentiment, and the mean topic proportion per time unit.

In addition, we examine the correlation between sentiments and traffic crash rates, data counts, and topics using Pearson correlation analysis. The correlation coefficient (*r*) is computed as follows:


$$r=\frac{\sum \left(X_{t}-\overset{-}{X}\right)\left(Y_{t}-\overset{-}{Y}\right)}{\sqrt{\sum {\left(X_{t}-\overset{-}{X}\right)}^{2}}\sqrt{\sum {\left(Y_{t}-\overset{-}{Y}\right)}^{2}}}$$

where $X_{t}$ (eg, negative emotions at quarter *t*) and $Y_{t}$ (eg, tweet count at quarter *t*) represent the paired values of the independent and dependent variables, respectively, and $\overset{-}{X}$ and $\overset{-}{Y}$ denote their mean values. A positive *r* indicates a positive correlation, while a negative *r* suggests an inverse relationship.

To quantify the proportion of variance explained, we also compute the coefficient of determination (*R*^2^) for regression models and the squared correlation coefficient (*r*^2^) for correlation analysis. Significance levels are calculated under the null hypotheses of $\beta_{1}=0$ for linear regression and r=0 for correlation analysis.

### Ethical Considerations

This study did not require institutional review board approval because it is an observational study that used only publicly accessible data and reported aggregate results with no personal identifiers. To maintain privacy and confidentiality, all personal identifiers were removed during data processing. Transparency was maintained throughout the study, with clear communication of its purpose, methods, and findings.

## Results

### Summary of Tweet Counts and Users

**Figure 3.**
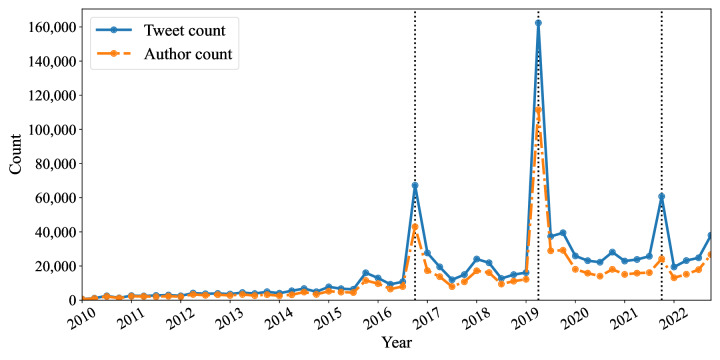
Tweet and user counts related to older drivers by quarter from 2010 to 2022. Black vertical dotted lines indicate the 3 major tweet peaks.

Our dataset contained 2,625,807 tweets from 1,052,976 unique users, comprising original tweets (n=767,419, 29.25%), retweets (n=1,678,133, 63.91%), replies (n=171,781, 6.54%), and quoted tweets (n=8474, 0.32%). To analyze primary opinions or original tweets, we excluded retweets and eliminated referenced text from quoted tweets and replies. Figure 3 illustrates the quarterly counts of original tweets and unique users discussing older drivers over a 13-year period. There has been a consistent presence of tweets and users, with a moderate increase over time. This trend is supported by linear regression (tweet count: $\beta_{0}$=−3011.09, $\beta_{1}$=832.75, *R*^2^=0.256, *P*<.001; user count: $\beta_{0}$=−1325.86, $\beta_{1}$=534.90, *R*^2^=0.238, *P*<.001).

Since 2016, the number of tweets per quarter has exceeded 10,000, rising to over 20,000 per quarter from 2019 onward. The highest peak occurred in the second quarter of 2019 (approximately 162,000 tweets), driven by a crash on April 19, 2019, in Higashi-Ikebukuro, Tokyo, where an 87-year-old man mistakenly stepped on the accelerator, killing a 3-year-old child and her mother and injuring 9 others [46]. The second peak, in the fourth quarter of 2016 (approximately 67,000 tweets), coincided with a series of crashes in Tochigi (November 10, 2016), Tokyo (November 12, 2016), and Miyazaki (November 13, 2016), where older drivers struck and killed pedestrians, sparking active public debate about older drivers [47]. Another significant peak in the fourth quarter of 2021 (approximately 60,000 tweets) followed a crash in Osaka, where an 89-year-old man mistakenly stepped on the accelerator, resulting in pedestrian casualties [48]. Hereinafter, we plot black dotted lines to indicate the 3 major tweet peaks in each figure.

### Sentiments Toward Older Drivers

After processing, our final dataset comprised 614,429 documents posted by 404,689 unique users. Table 1 presents the proportion of documents containing words associated with sentiments in the primary emotional subcategories (“affective processes” and “negative emotions”) as well as “drives.”

Across all quarters, negative emotions (n=383,520, 62.42%) were more prevalent than positive emotions (n=217,300, 35.37%) in “affective processes.” Within the subcategory of “negative emotions,” anxiety (n=114,234, 18.59%) and anger (n=106,767, 17.38%) were more frequent than sadness (n=23,609, 3.84%). In “drives,” risk (n=357,311, 58.15%) was the most frequently observed sentiment. Sentiments in other categories, none of which exceeded an average proportion of 50%, are presented in “S2. Sentiment analysis Details” in Multimedia Appendix 1. For instance, family (n=65,069, 10.59%) in “social processes,” insights (n=261,040, 42.48%) in “cognitive processes,” and health (n=80,967, 13.18%) in “biological processes” were the most predominant sentiments in their respective categories.

**Figure 4.**
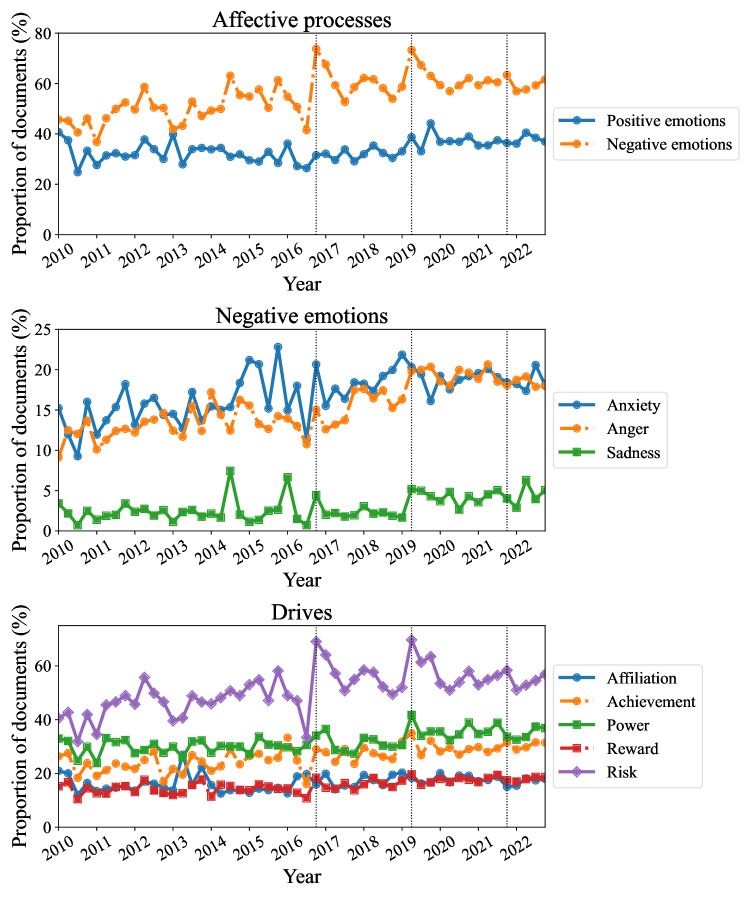
The Japanese version of the Linguistic Inquiry and Word Count Dictionary quarterly trends in “affective processes,” “negative emotions,” and “drives.” Black vertical dotted lines indicate the 3 major tweet peaks as illustrated in Figure 3.

Temporal trends of sentiments are plotted in Figure 4, with the numeric results of linear regression presented in Table 1. Regarding “affective processes,” while positive emotions shows a slight increase ($\beta_{1}$=0.11, *P*=.004), negative emotions increased more prominently by 0.36% per quarter (*P*<.001), rising from approximately 40% to 60%, with spikes aligning with the 3 previously discussed data peaks, as indicated by black dotted lines. All sentiments in “negative emotions” display an increasing trend, with anxiety (n=114,234, 18.59%, $\beta_{1}$=0.12%, *P*<.001) and anger (n=106,767, 17.38%, $\beta_{1}$=0.17%, *P*<.001) showing relatively strong upward trends compared with sadness (n=23,609, 3.84%, $\beta_{1}$=0.05%, *P*<.001). Regarding “drives,” risk remained consistently above 40% and showed a noticeable increase over time ($\beta_{1}$=0.33%, *P*<.001) relative to other sentiments. Additional analyses can be found in “S2. Sentiment Analysis Details” in Multimedia Appendix 1, indicating that insights (n=261,040, 42.48%, $\beta_{1}$=0.25%, *P*<.001) and hear (n=87,281, 14.21%, $\beta_{1}$=0.19%, *P*<.001) were relatively dominant and exhibited increasing trends.

### Topics About Older Drivers

Topic modeling with 2 layers of NMF identified 29 dynamic topics that achieved the highest coherence score of 0.22 in an experiment varying the number of topics between 25 and 90 (Multimedia Appendix 1).

We further selected 16 topics that constituted at least 3.5% of all documents on average, naming them based on the top-weighted words and representative original tweets for each topic. Finally, we selected 9 topics where the *R*^2^ value exceeded 0.1 in simple linear regression, represented by a red dashed line, as illustrated in Table 2. The details of all 16 topics, including typical tweets (in both Japanese and English), as well as the regression results for the 7 topics whose *R*² did not exceed 0.1, are provided in Multimedia Appendix 1.

**Table 2.** Description of 9 topics, including topic names, top 10 weighted words, proportions, and results of linear regression (the formula $Y_{t}=\beta_{0}+\beta_{1}X_{t}+\epsilon_{t}$).

Topic name	Top 10 weighted words	Tweets, n (%)	$\beta_{0}$	$\beta_{1}$	*R* ^2^	*P* value
Topic 1: License surrender	return, license, voluntary, revoke, system, forcefully, surrender, certificate, revocation, discount	43,270 (7.04)	4.37	0.08	0.278	<.001
Topic 2: Crash events	crash, cause, increase, traffic, report, death, decrease, frequent, occur, prevention	35,396 (5.76)	2.73	0.07	0.358	<.001
Topic 5: Traffic safety	traffic, safety, campaign, prevention, bicycle, drinking, nationwide, walking, public, child	25,139 (4.09)	6.13	−0.05	0.123	.010
Topic 6: Ikebukuro incident	runaway, Ikebukuro, Tokyo, NHK, defendant, crash, bereaved, family, memorial*^a^*	24,430 (3.98)	−0.10	0.09	0.343	<.001
Topic 7: Self-driving technology	self-driving, social, Japan, technology, necessary, safety, soon, experiment, popularization, development	24,235 (3.94)	1.95	0.05	0.151	.004
Topic 8: Social issues	issue, social, consider, life, rural, solution, necessary, difficult, traffic, local	23,935 (3.90)	1.57	0.05	0.294	<.001
Topic 9: License renewal	renewal, license, course, take, exam, test, center, excellent, Emperor, Kobe	23,525 (3.83)	5.83	−0.05	0.126	.010
Topic 10: Discussing senior driving	say, bad, hear, go, oneself, come, same, can, young, complain	23,511 (3.83)	1.30	0.07	0.402	<.001
Topic 14: Driving errors	brake, accelerator, step, mistake, wrong, pedal, crash, parking, MT^b^, operation	21,741 (3.54)	2.23	0.04	0.199	<.001

a The frequently mentioned name of the individual involved in this crash has been masked to consider ethical concerns.

b MT indicates Manual Transmission, which is one type of vehicle transmission system.

The most prevalent topics were License surrender (n=43,270, 7.04%) and Crash events (n=35,396, 5.76%), followed by Traffic safety, Ikebukuro incident, Self-driving technology, Social issues, License renewal, Discussing senior driving, and Driving errors. Other topics include Thoughts on older drivers, Prevalent older drivers*,* and News media (“S3. Topic Modeling Details” in Multimedia Appendix 1).

**Figure 5.**
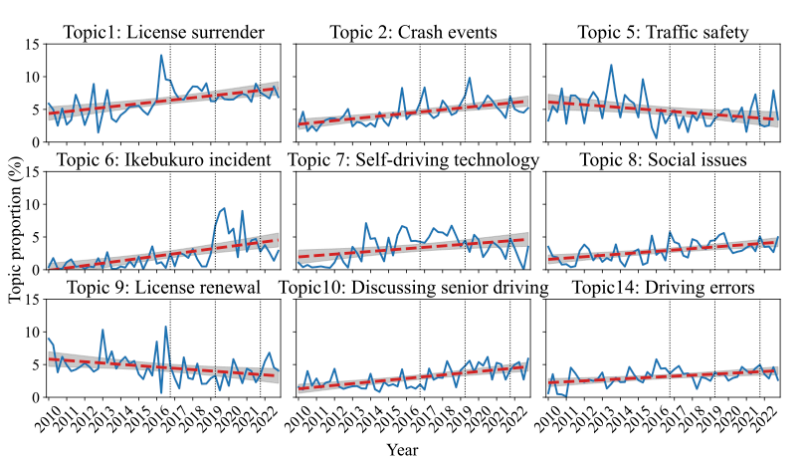
Temporal trends of 9 topic mean proportions, shown as solid blue lines, with red dashed lines representing regression lines. Gray shaded areas represent 95% CIs for the regression lines. Black vertical dotted lines indicate the 3 major tweet peaks as illustrated in Figure 3.

Figure 5 illustrates the temporal trends of topic proportions for 9 topics with a red dashed line, representing the simple linear regression result for each topic. The proportions of topics 1 (License surrender, $\beta_{1}$=0.08, *P*<.001), 2 (Crash event, $\beta_{1}$=0.07, *P*<.001), 6 (Ikebukuro incident, $\beta_{1}$=0.09, *P*<.001), 8 (Social issues, $\beta_{1}$=0.05, *P*<.001), 10 (Discussing senior driving, $\beta_{1}$=0.07, *P*<.001) and 14 (Driving errors, $\beta_{1}$=0.04, *P*<.001) show consistent increases over time. Notably, topic 1 peaked in 2016, when measures to promote license surrender in rural areas gained attention. Topics 2 and 8 correspond to tweet count peaks, while topic 6 coincides specifically with the fourth quarter of 2019 subsequent periods. Although less pronounced, topic 7 (Self-driving technology, $\beta_{1}$=0.05, *P*=.004) also exhibits an increasing trend, whereas topics 5 (Traffic safety, $\beta_{1}$=−0.05, *P*=.01) and 9 (License renewal, $\beta_{1}$=−0.05, *P*=.001) are declining. Additional analysis can be found in “S3. Topic Modeling Details” in Multimedia Appendix 1). Notably, News media exhibits topic peaks in the fourth quarter of 2016, reflecting unique characteristics not captured by simple linear regression.

### Correlation Analysis on Sentiments

Table 3 and Table 4 present the results of the correlation analysis for positive emotions and negative emotions in “affective processes,” risk in “drives” and anxiety, anger, and sadness in “negative emotions,” across 4 crash rates, 2 data counts, and 9 topics. While the correlation analysis for crash rates among individuals aged more than 70 years and 80 years is based on yearly data (n=13) due to the limited availability of statistical data [14], the other analyses are based on quarterly data (n=52).

**Table 3.** Results of the correlation analysis for positive emotions, negative emotions, and risk.

Variables	Positive emotions			Negative emotions			Risk		
	*R*	*r* ^2^	*P* value	*R*	*r* ^2^	*P* value	*R*	*r* ^2^	*P* value
Crash rate (n=13)									
16‐29	–0.747	0.559	.003	–0.750	0.563	.003	–0.702	0.493	.007
30‐59	–0.714	0.510	.006	–0.784	0.615	.002	–0.739	0.546	.004
60‐69	–0.687	0.473	.009	–0.799	0.638	.001	–0.754	0.568	.003
Over 70	–0.712	0.507	.006	–0.787	0.619	.001	–0.744	0.554	.004
Data count (n=52)									
Tweet count	0.317	0.101	.02	0.683	0.466	<.001	0.677	0.459	<.001
User count	0.311	0.097	.03	0.674	0.454	<.001	0.671	0.450	<.001
Topic (n=52)									
Topic 1	0.039	0.002	.78	0.396	0.157	.004	0.364	0.132	.008
Topic 2	0.173	0.030	.22	0.761	0.579	<.001	0.719	0.517	<.001
Topic 5	–0.037	0.001	.79	–0.173	0.030	.220	–0.111	0.012	.430
Topic 6	0.425	0.180	.002	0.546	0.298	<.001	0.554	0.306	<.001
Topic 7	0.003	0.000	.98	0.351	0.123	.010	0.381	0.146	.005
Topic 8	0.270	0.073	.05	0.714	0.510	<.001	0.693	0.480	<.001
Topic 9	0.217	0.047	.12	–0.496	0.246	<.001	–0.526	0.277	<.001
Topic 10	0.264	0.069	.06	0.425	0.181	.002	0.365	0.133	.008
Topic 14	0.177	0.031	.21	0.43	0.185	.001	0.41	0.168	.003

**Table 4.** Results of the correlation analysis for anxiety, anger, and sadness.

Variables	Anxiety			Anger			Sadness		
	*R*	*r* ^2^	*P* value	*R*	*r* ^2^	*P* value	*R*	*r* ^2^	*P* value
Crash rate (n=13)									
16‐29	–0.754	0.568	.003	–0.938	0.881	<.001	–0.742	0.550	.004
30‐59	–0.795	0.631	.001	–0.925	0.856	<.001	–0.744	0.554	.004
60‐69	–0.818	0.669	<.001	–0.913	0.833	<.001	–0.743	0.551	.004
Over 70	–0.788	0.620	.001	–0.920	0.847	<.001	–0.735	0.540	.004
Data count (n=52)									
Tweet count	0.409	0.168	.003	0.519	0.269	<.001	0.442	0.196	.001
User count	0.407	0.166	.003	0.516	0.266	<.001	0.438	0.192	.001
Topic (n=52)									
Topic 1	0.419	0.175	.002	0.297	0.088	.030	0.158	0.025	.260
Topic 2	0.526	0.277	<.001	0.548	0.300	<.001	0.380	0.144	.005
Topic 5	–0.121	0.015	.390	–0.186	0.035	.190	–0.132	0.017	.350
Topic 6	0.375	0.141	.006	0.677	0.459	<.001	0.512	0.262	<.001
Topic 7	0.291	0.085	.040	0.235	0.055	.090	0.000	0.000	≥.99
Topic 8	0.497	0.247	<.001	0.485	0.235	<.001	0.396	0.157	.004
Topic 9	–0.514	0.264	<.001	–0.386	0.149	.005	–0.028	0.001	.840
Topic 10	0.385	0.148	.005	0.682	0.465	<.001	0.287	0.082	.040
Topic 14	0.246	0.061	.080	0.258	0.066	.070	0.366	0.134	.008

For the crash rate of individuals aged between 60 and 69 years, negative emotions (*r*=−0.80, *P*=.001) and anxiety (*r*=−0.82, *P*<.001) exhibit strong negative correlations, similar to those observed for individuals aged more than 70 years. Reversely, anger exhibits the strongest negative correlation with the youngest age group (16-29 y). Regarding the tweet count, negative emotions (*r*=0.68, *P*<.001), risk (*r*=0.68, *P*<.001), and anger (*r*=0.52, *P*<.001) exhibit moderate to strong positive correlations, similar to the user count.

For topics, topics 2 (Crash events, *r*=0.76, *P*<.001), 6 (Ikebukuro incident, *r*=0.55, *P*<.001), 8 (Social issues, *r*=0.71, *P*<.001), and 9 (License renewal, *r*=−0.50, *P*<.001) are significantly correlated with negative emotions, similar to risk. Furthermore, while positive emotions do not show significant correlations with any topic, other sentiments are correlated with multiple topics: anxiety with topics 2, 8, and 9; anger with topics 2, 6, 8, and 10 (Discussing senior driving); and sadness with topic 6.

## Discussion

### Principal Findings

In this study, we conducted a quantitative analysis of discourse regarding older drivers from 2010 to 2022 in Japan using Twitter. The number of tweets and users discussing older drivers has increased since 2016, with peaks observed in 2016, 2019, and 2021. Sentiment analysis revealed that negative emotions were more prevalent and increased over time compared with positive emotions. In addition, contexts related to anxiety, anger, and risk were prevalent and showed an upward trend. Topic modeling identified themes primarily related to driving licenses, crash events, personal perspectives, and traffic issues. Chronologically, despite decreasing trends in Traffic safety and License renewal, topics such as Crash events and License surrender are increasing. Finally, correlation analysis revealed that negative emotions were negatively correlated with crash rates among older drivers, positively correlated with tweet counts, and positively associated with topics such as Crash events and Ikebukuro incident.

This study quantified issues surrounding older drivers through Twitter, a leading social media platform, contributing to advancements in research on public health and ageism. The function of social media, such as posting and sharing tweets on any subject along with figures and URLs in Twitter, enables to prompt the swift dissemination of information [49] and derive population-level inferences. These have fostered new fields in public health sectors such as infoveillance [50], digital epidemiology [51], and digital disease detection [52]. They also led to an increase in health-related studies in Japan including hospitals [53], disease information [54], and eHealth literacy [55]. In addition to public health, the issue of older drivers has the aspects of ageism, which encompass our thoughts (stereotypes), emotions (prejudice), and behaviors (discrimination) toward others based on age [56]. Ageism toward older people is especially high [57] and can adversely affect physical and mental health, such as reduced cognitive function [58], shorter life expectancy [59], deteriorated mental health [60], and increased isolation [61], prompting initiatives to counteract it [62]. Research on ageism is seen across multiple domains including health research [63], mental health services [64], long-term care facilities [65,66], workplaces [67,68], and media representations [69-71], with recent Twitter-based approaches employing thematic [72-76], descriptive [75,76], computational modeling [77,78], and quantitative text analyses [79-82]. This study, therefore, addresses the issues surrounding older drivers from both public health and ageism perspectives, contributing insights toward mitigating these challenges.

An abrupt increase in tweet counts sporadically observed over time is likely attributable to high-profile crashes caused by older drivers. The highest peak in the second quarter of 2019 corresponding to topic 6 (Ikebukuro incident) coincided with a crash that occurred in Higashi-Ikebukuro, Tokyo, drawing significant media and online attention for several months due to widespread sadness and anger toward the man, leading to the highest recorded number of driver’s license surrenders in 2019 [2]. The peak in the fourth quarter of 2016, associated with topic 8 (Social issues) and topic 11 (News media) coincided with a series of crashes, and the peak in the fourth quarter of 2021, followed by a crash in Osaka, led to an increase in tweets related to topic 2 (Crash events) and topic 14 (Driving errors).

Regarding sentiments, all 3 peaks coincided with peaks in negative emotions and risk, with average proportions remaining somewhat higher after the peaks than before, while positive emotions were less relevant. Thus, the data peaks of tweets about older drivers are largely facilitated by contexts of negative emotions and risks, supported by the trends in their specific crashes aggregated by topics.

The detected topics help us understand how society forms opinions and perceptions toward older drivers in the long run. Among the 16 topics accounting for over 3.5%, we identified 7 increasing topics and 2 decreasing topics. Driving licenses are discussed in both topics 1 and 9, with public discourse increasingly focusing on license surrender (topic 1 is rising) rather than renewal (topic 9 is declining), suggesting a social shift toward discouraging older adults from driving and a potential impact on the number of license surrenders [2]. Topic 2 (Crash events), including tweets such as “It seems that crashes involving older drivers are occurring frequently” and “Let’s prevent crashes involving older drivers,” reflects general discussions about such events, often triggered by specific incidents, as seen in data peaks in 2016 and 2019. In contrast, topic 6 (Ikebukuro incident) includes a summary of the Ikebukuro incident and the perpetrator [46], showing a notable increase in 2019, and topic 14 (Driving errors) highlights that crashes caused by older drivers often occur due to physical or operational limitations, as shown in meta-review [4]. Over the data collection period, topic proportions shifted from topic 5 (Traffic safety; prominent until 2015) and topic 7 (Self-driving technology; high between 2013 and 2019) to topics 8 (Social issues) and 10 (Discussing senior driving), both of which have recently increased. These topics express concerns about older drivers from societal (topic 8) and family (topic 10) perspectives. Other recurring topics include Thoughts on older drivers, Prevalent older drivers, and News media, which consistently appear in this discourse. Notably, topic 11 (News media), which includes references to major media outlets, showed a high proportion between 2016 and 2017, suggesting a potential media overemphasis on older driver–related issues. (Example tweets for these and other topics not discussed in the main text are provided in “S3. Topic Modeling Details” in Multimedia Appendix 1).

Our analysis clarifies the sentiments in public discourse and their correlations, particularly negative emotion, anxiety, anger, and risk, which have shown a persistent and increasing trend over time. The proportion of documents expressing negative emotions rose from approximately 40% to 60%, accounting for an increase of 1.4% per year. This trend is strongly positively correlated with data count and with topics 2 (Crash events), 6 (Ikebukuro incident), and 8 (Social issues). In contrast, negative emotions are most significantly and strongly negatively correlated with the crash rates of older adults (aged 60-69 y and 70+ y; *P*=.001), although similar trends are also observed in other age groups. These suggest that negative perceptions toward older drivers arise from heightened attention to their crash events and recognition of the issue as a social problem. Similar to negative emotions, the sentiment of risk increased from 40% to 60% (1.3% per year), with peaks around 70% in 2016 and 2019. This indicates that public perception includes not only negativity toward older drivers but also a sense of danger regarding their driving. Within “negative emotions,” anxiety and anger, each accounting for approximately 20%, are more prevalent than sadness. Although they do not show sharp peaks, both have steadily increased over time. While their correlation patterns are similar to those of negative emotions, stronger correlations are observed; the negative correlation between anxiety and topic 9 (License renewal) suggests decreasing focus on continued driving, while the positive correlation between anger and topic 10 (Discussing senior driving) indicates emotional overrepresentation of older drivers in discourse. Positive emotions show no significant correlation with any of the variables. Sadness, however, is correlated with topic 6 (Ikebukuro incident), suggesting compassion for the mother and child who were victims in the incident. Overall, driving by older people is increasingly perceived as negative and risky, and this perception is influenced by strong negative emotions such as anxiety and anger, which amplify public discourse on the topic despite a declining trend in their actual crash rates.

Finally, we present our perspectives. First, negative stereotypes and prevailing public sentiment toward older drivers may influence licensing policies [3]. Our analysis reveals contrasting trends between topics related to license renewal and license surrender, suggesting a shift in public perception toward encouraging older individuals to stop driving. However, this shift tends to overlook the potential adverse effects of driving cessation, as noted in previous studies [5-7]. Although analysis of the relationship between public discourse and licensing policies remains limited, it is essential to reconsider current policies that may discourage older people from driving—such as overly strict licensing requirements [4,5] and campaigns promoting license surrender [2]—in favor of approaches that both reduce traffic crashes and ensure their safety.

Second, the disproportionate public attention given to traffic crashes involving older drivers is a significant concern. Although the anger directed at tragic crashes involving older drivers is understandable, many older individuals follow traffic laws and drive responsibly [4], therefore it is unfair that the entire older population faces negative consequences as a result. To address this, it is necessary to implement risk-based licensing policies that focus on high-risk individuals, as well as to promote autonomous driving technologies and infrastructure that support continued safe mobility for older adults.

Third, public debate following crashes involving older drivers often centers on specific high-profile incidents rather than on the overall frequency or statistical context of such crashes. This may be partly due to media tendencies to sensationalize older driver involvement, as seen in the temporary increase in topic 11 (News Media). Media use influences the formation and stability of public opinion clusters [83] and can contribute to heightened public anxiety during periods of uncertainty, such as pandemics [84]. Providing the public with a more accurate and balanced understanding of older drivers—including countering media-driven bias—is crucial to fostering constructive discourse. This may involve encouraging more responsible media reporting and promoting public access to objective information on older driver safety.

In summary, this study offers a long-term analytical approach that integrates sentiment and textual content, contributing to research on public discourse related to aging issues and, more broadly, public health. Our findings enhance the understanding of societal perceptions and policy implications surrounding older drivers in Japan and may inform future discussions on aging societies around the world.

### Limitations

Our study had several limitations. First, while Twitter is one of the most widely used social media platforms in Japan [85], Twitter users do not necessarily represent the general population [86]. Furthermore, Twitter provides access only to tweets from public accounts, deterring extreme or controversial textual content to be tweeted or we might have missed such tweets because past tweets can be deleted. In addition, display algorithms were modified around 2011 due to a rapid increase in Twitter users, resulting in nonstationarities in the dataset. To mitigate such user biases, several approaches have been proposed, including evaluating users based on the authenticity and bias of their engagements [87], and accounting for population demographics and word ambiguity [88].

Second, while the keyword (“(高齢 OR 老人) AND (運転 OR ドライバー)” in Japanese) effectively targets discussions about older drivers, there is still room for refinement. “運転” (driving) and “ドライバー” (driver) are the words related to driving, representing specifically older driving by combing words “高齢” (denoting old age) or “老人” (denoting older people). Although adding “事故” (crash) was considered, this word could collect data unrelated to crashes like falls in daily life, without words related to driving. Other age-related words like “80歳のドライバー” (80-year-old drivers) or “祖父” (grandfather) specify too detailed ages or familial relations, which are less general and more restrictive, so we did not use them aligning with our research objectives. In addition, we found that a small portion (33 out of 1000) of the sampled tweets were not related to older drivers but represented older people in nondriving contexts such as riding the bus or being crash victims. Therefore, while the data from this study appears to be sufficiently valid, there is a need to refine the keyword or explore other methods to collect more accurate data on older drivers.

Third, our methods have room for improvement. Word-based approaches for sentiment analysis and topic modeling offer valid and intuitive interpretations for emerging themes such as older driver–related discourse, but they may not fully capture contextual nuances such as negations or compound expressions. Although the J-LIWC dictionary covered 48% (4458 out of 9287) of the words used in this study—indicating substantial coverage with room for refinement—our findings remain persuasive, especially when compared with the Japanese version of the Moral Foundations Dictionary [89], which covers only 3% (275 words) (“S3. Sentiment Analysis Details” in Multimedia Appendix 1). Furthermore, the coherence score for the dynamic topic model was 0.22, lower than the commonly accepted baseline of 0.36 [36]. This may be influenced by the linguistic characteristics of Japanese and the use of a Japanese word2vec model. Finally, as correlation analysis does not imply causation, more rigorous statistical testing is needed. Therefore, although our word-based method using reliable dictionaries offers high interpretability, future work should explore alternative methods—such as deep-learning–based sentiment analysis or BERTopic for topic modeling—and incorporate techniques like supervised machine learning or community detection to gain deeper insights [24,41,90,91].

### Conclusions

Despite the actual decline in crash rates among older drivers, previous surveys in Japan reveal persistent negative sentiments toward them, suggesting the need to understand discourse on social media, which serves as a platform for public debate. To quantitatively assess public awareness, we collected and analyzed tweets related to older drivers. The results revealed an increase in the number of tweets from 2010 to 2022, with certain peaks aligning with heightened attention to crashes involving older drivers. Negative emotions and risk consistently displayed high and rising levels, primarily correlated with the topic of crash events. Furthermore, there are diverse topics related to drivers’ licenses, crash events, and traffic safety. These imply unfair public recognition toward older drivers, suggesting the need for reconsidering current license policies, promoting self-driving systems, and facilitating accurate and balanced understanding, in order to provide continued safe mobility for older adults.

## Supplementary material

10.2196/69321 Multimedia Appendix 1 Supplementary details of the analysis.

## Abbreviations

DTM: dynamic topic model
J-LIWC: Japanese version of the Linguistic Inquiry and Word Count Dictionary
NMF: nonnegative matrix factorization
