# Supplementary material for: Public Discourse Toward Older Drivers in Japan Using Social Media Data From 2010 to 2022: Longitudinal Analysis
Source: JMIR Infodemiology. 2025 Jun 16;5:e69321. doi: 10.2196/69321 (PMC12209477; doi:10.2196/69321)
Supplement: Multimedia Appendix 1 [file infodemiology-v5-e69321-s001.docx]

# Multimedia Appendix 1. Supplementary details of the analysis.

## S1. Stopwords list

1. Words used in the query: [“高齢”, “老人”, “運転”, “ドライバー”]

2. Words that are only in Japanese Hiragana

3. One-letter words

4. Numbers-only words

5. A predefined stopword list based on the SlothLib Project [1], which is commonly used for Japanese natural language processing. This list includes general-purpose words that do not contribute significantly to the meaning of a sentence, such as:
[“ハイ” (yes), “下記” (below), “上記” (above), “ヶ所” (location), “カ所” (location), “箇所” (location), “ヶ月” (months), “今回” (this time), “前回” (last time), “場合” (case/situation), “一つ” (one), “年生” (grade/year), “以前” (before), “以後” (after), “以降” (thereafter), “未満” (less than), “以上” (more than), “以下” (less than or equal to), “幾つ” (how many)].

## S2. Sentiment Analysis Details

### S2.1 Results of Sentiment Analysis in J-LIWC

We show the trends in the proportion of categories not discussed in the main text (Table S1, Figure S1 and S2), but we omitted Informal language, which has a low ratio.

Table S1. Proportion of documents containing words corresponding to 20 sentiments in the J-LIWC’s “social processes”, “cognitive processes”, “perceptual processes” and “biological processes” with proportions and results of linear regression (the formula$Y_{t}= \beta_{0}+\beta_{1}X_{t}+\epsilon_{t}$).

| Subcategory | Sentiment | Proportion | $\beta_{0}$ | $\beta_{1}$ | *R^2^* | *P* value |
| --- | --- | --- | --- | --- | --- | --- |
| Social processes | *Family* | 10.6 | 7.8 | 0.07 | 0.184 | 0.002 |
|  | *Friend* | 3.1 | 3.1 | 0.00 | 0.000 | 0.879 |
|  | *Female references* | 5.5 | 4.9 | 0.02 | 0.059 | 0.083 |
|  | *Male references* | 5.6 | 3.9 | 0.04 | 0.142 | 0.006 |
| Cognitive processes | *Insights* | 42.5 | 32.1 | 0.25 | 0.552 | <.001 |
|  | *Causation* | 20.4 | 15.9 | 0.09 | 0.281 | <.001 |
|  | *Discrepancy* | 11.1 | 7 | 0.10 | 0.351 | <.001 |
|  | *Tentative* | 13.9 | 10.4 | 0.08 | 0.312 | <.001 |
|  | *Certainty* | 21.3 | 15.2 | 0.13 | 0.437 | <.001 |
|  | *Differentiation* | 11.6 | 7.2 | 0.10 | 0.534 | <.001 |
| Perceptual processes | *See* | 13.4 | 11.8 | 0.04 | 0.106 | 0.019 |
|  | *Hear* | 14.2 | 10.5 | 0.19 | 0.342 | <.001 |
|  | *Feel* | 5.9 | 4.4 | 0.04 | 0.269 | <.001 |
| Biological processes | *Body* | 2.5 | 3.1 | -0.01 | 0.106 | 0.019 |
|  | *Health* | 13.2 | 11.5 | 0.04 | 0.066 | 0.065 |
|  | *Sexual* | 0.0 | 0.2 | -0.00 | 0.097 | 0.024 |
|  | *Ingestion* | 1.0 | 1.7 | -0.02 | 0.069 | 0.059 |
| Relatively | *Motion* | 44.6 | 41.5 | 0.12 | 0.142 | 0.006 |
|  | *Time* | 39.2 | 38.2 | 0.06 | 0.045 | 0.13 |
|  | *Space* | 47.4 | 43.8 | 0.07 | 0.066 | 0.066 |


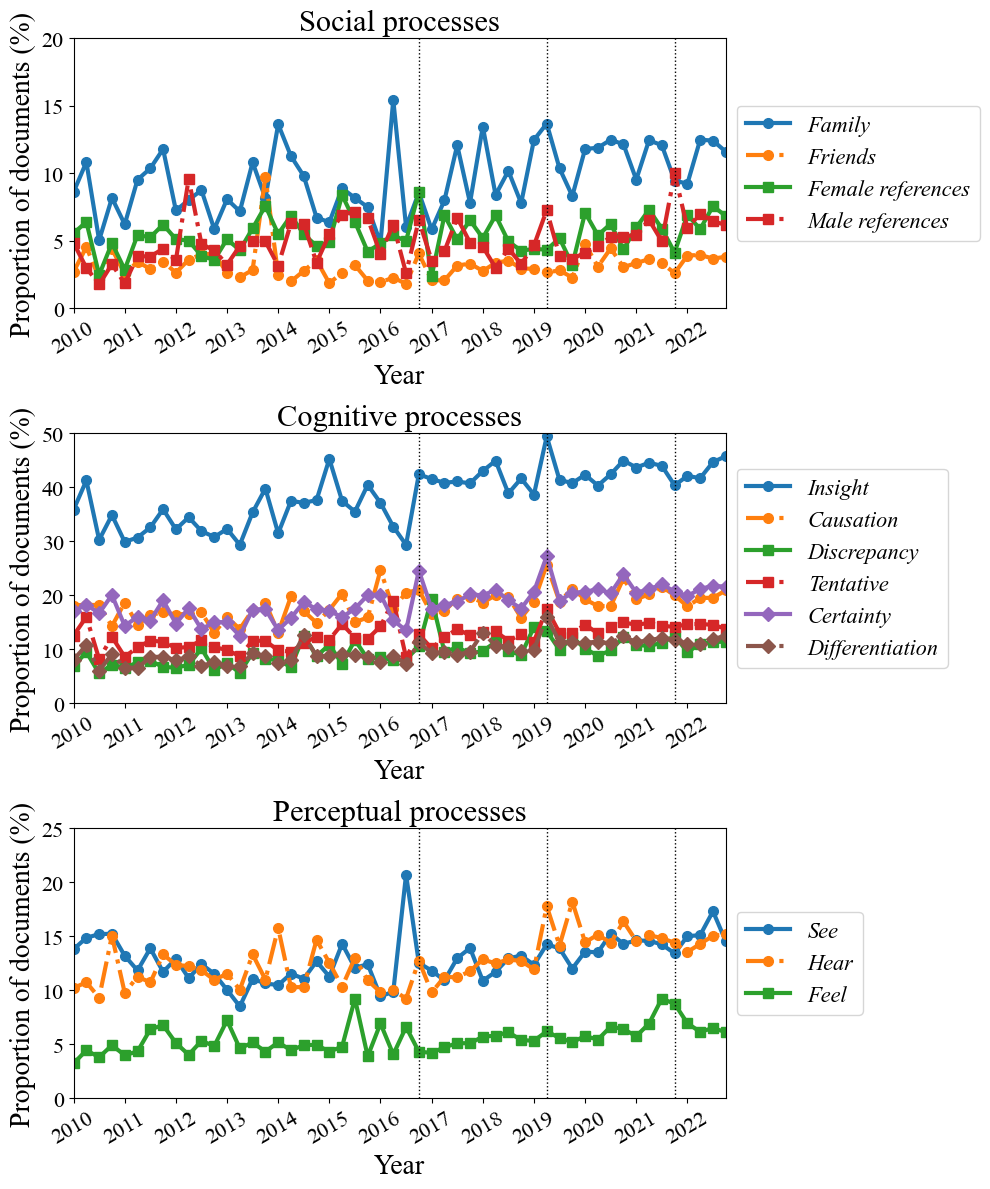


Figure S1: J-LIWC quarterly trends (“social Processes”, “cognitive processes” and “perceptual processes”)
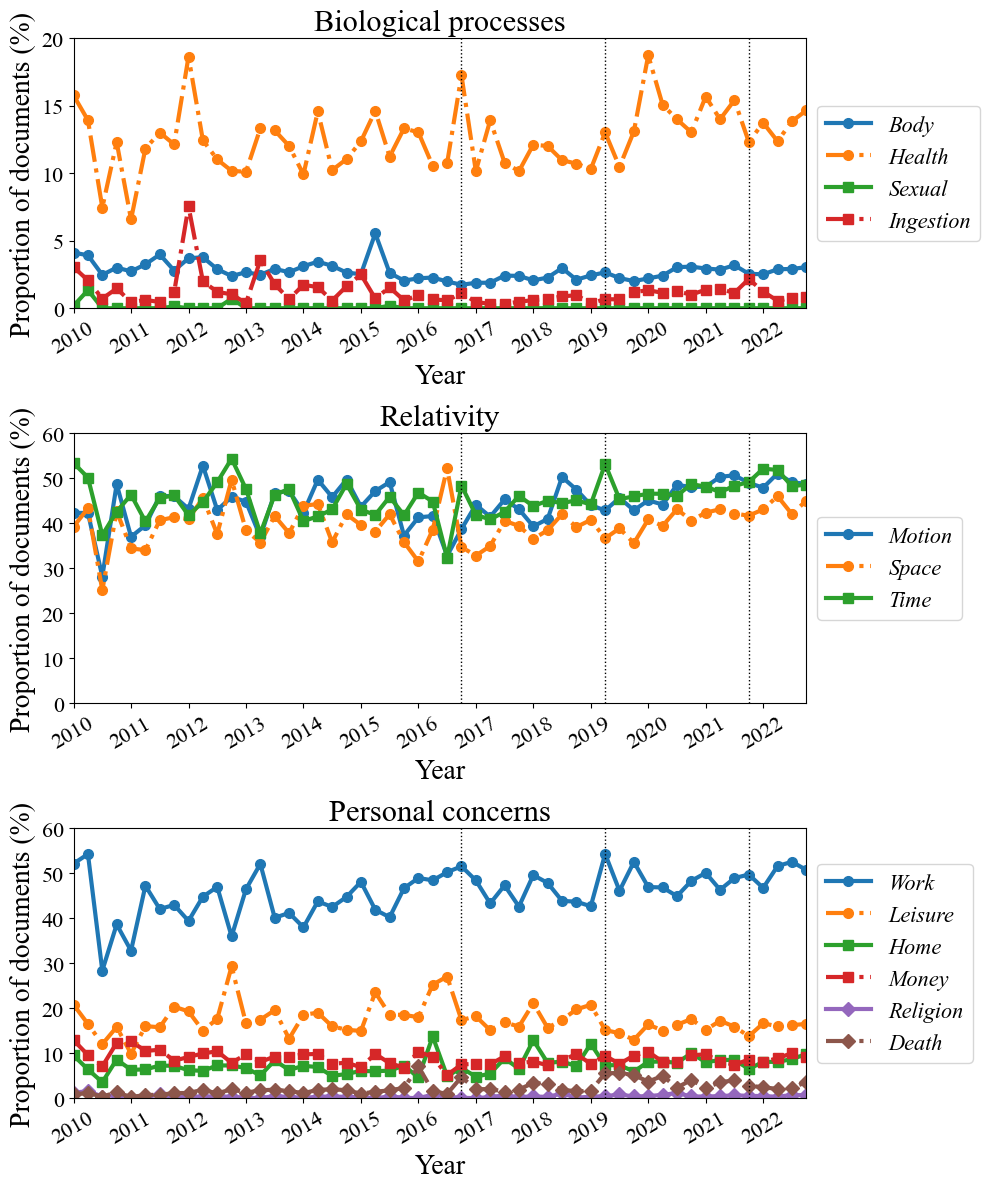


Figure S2: J-LIWC quarterly trends (“biological processes, “relativity” and “personal concerns”)

### S2.2 Results of Sentiment Analysis in J-MFD

As a way of comparing with the J-LIWC, we also used the Japanese version of Moral Foundations Dictionary (J-MFD) [2]. The J-MFD mainly comprises 10 sentiments: five moral foundations, namely “harm”, “fairness”, “ingroup”, “authority” and “purity” with two dimensions (“virtue” or “vice”) in each foundation. Even though we include stem matching words following to proposed paper (indicated by an asterisk) [2], in addition to perfect matches for the J-LIWC, only 275 words (3.0%) are seen in words on our dataset. This is influenced by the small originally containing number of words (714 words in J-MFD), and this is the reason to omit results in J-MFD from main text.

Table S2 shows the proportions and results of linear regression in J-MFD, suggesting the sentiments in the J-MFD were generally less prevalent than those in the J-LIWC but the most prevalent sentiment in the J-MFD was *harm virtue* (10.7%), which focuses on protecting victims of traffic crashes. Following this, *ingroup virtue* (9.6%) and *authority virtue* (8.7%) also appear frequently; the former relates to contexts involving family or society, while the latter arises from policies and regulations concerning driving licenses. Quarterly trends in J-MFD are also shown in Figure S3 and S4.

Table S2. Proportion of documents containing words corresponding to 5 foundations with 2 dimensions in J-MFD with proportions and results of linear regression (the formula$Y_{t}= \beta_{0}+\beta_{1}X_{t}+\epsilon_{t}$).

| Foundation | Dimension | Proportion | $\beta_{0}$ | $\beta_{1}$ | *R^2^* | *P* value |
| --- | --- | --- | --- | --- | --- | --- |
| *Harm* | *Virtue* | 10.68 | 9.57 | 0.03 | 0.039 | 0.163 |
|  | *Vice* | 6.65 | 3.39 | 0.07 | 0.263 | <.001 |
| *Fairness* | *Virtue* | 1.08 | 1.01 | 0 | 0.002 | 0.779 |
|  | *Vice* | 3.19 | 2.21 | 0.03 | 0.415 | <.001 |
| *Ingroup* | *Virtue* | 9.62 | 7.78 | 0.04 | 0.1 | 0.022 |
|  | *Vice* | 1.85 | 0.93 | 0.02 | 0.322 | <.001 |
| *Authority* | *Virtue* | 8.71 | 6.1 | 0.06 | 0.291 | <.001 |
|  | *Vice* | 2.73 | 1.73 | 0.03 | 0.204 | <.001 |
| *Purity* | *Virtue* | 0.25 | 0.29 | 0 | 0.033 | 0.198 |
|  | *Vice* | 2.85 | 1.07 | 0.05 | 0.348 | <.001 |


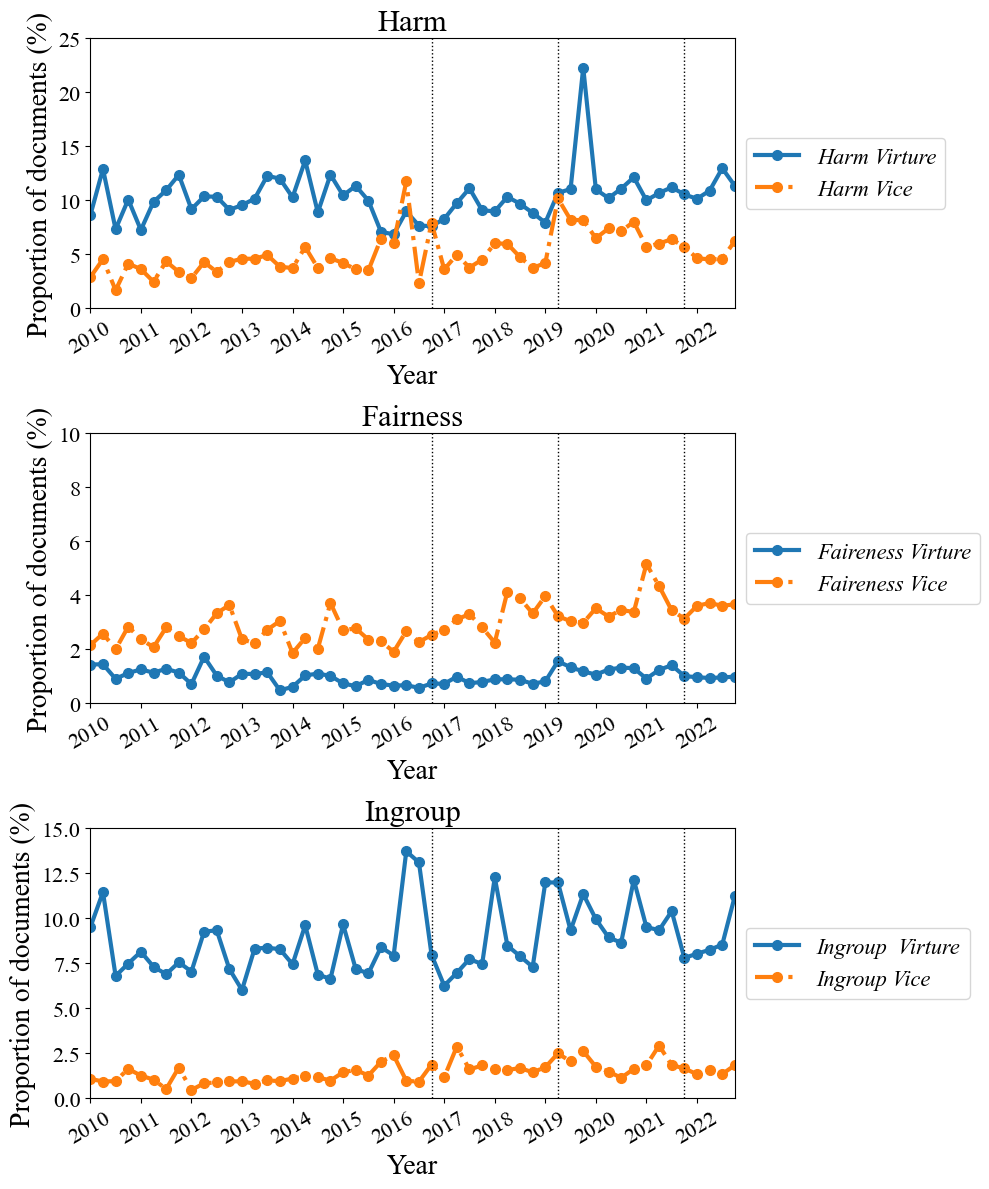


Figure S3: J-MFD quarterly trends (*harm*, *fairness* and *ingroup*)


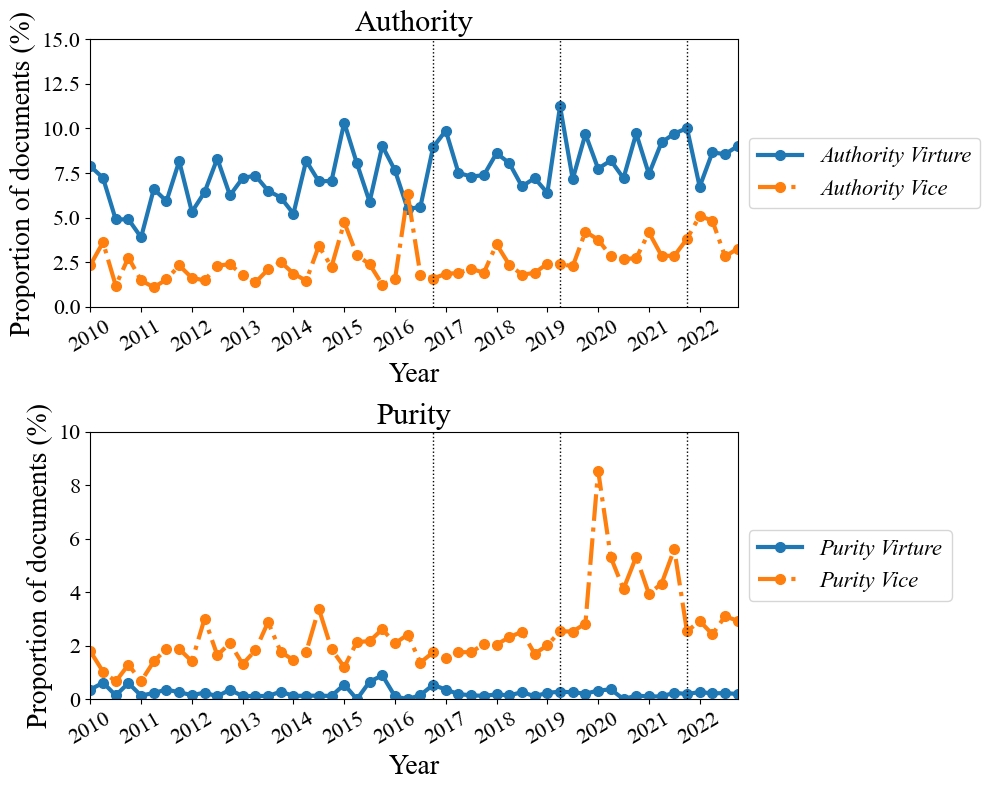


Figure S4: J-MFD quarterly trends (*authority* and *purity*)

## S3. Topic Modeling Details

### S3.1 Setting of Two Layers of NMF

This chapter explains detail steps of two layers of NMF following submitted paper [3]. NMF is an unsupervised method [4] for dimensionality reduction of non-negative matrices, aiming to decompose the data into factors that are constrained to contain only non-negative values. Two layers of NMF consist of the following:

1. **Prepare for 1st layer**: After applying tf-idf transformation and document length normalization for each quarter, we obtain 52 document-term matrices $A_{t}\in R^{n\times m}$(t=2010−Q1, 2010−Q2, …, 2022−Q4). *m* means a number of words with an occurrence rate of 0.1% or more for each quarter (see “Data collection and Processing” in main text).
2. **1st layer of NMF**: We apply NMF to all $A_{t}$ using the number of window topics $k_{t}\in[10, 25]$, and we obtain window topic distributions for each document.
3. **Prepare for 2nd layer**: To blend the results of the 1st layer, we pick up $m^{'}\in\{10,15, 20\}$ words from them and obtain the (window) topic-term matrix $B\in R^{n\times m}$.
4. **2nd layer of NMF**: We apply NMF to $B$ using the number of dynamic topics $k^{'}$, and we obtain dynamic topic distributions for each document.

To specify parameters, we use TC-W2V coherence (using default setting top 10 words to evaluate) [5] using large-scale Japanese Social Media Corpus [6]. It is expressed by approximately 2 million Japanese words from SNS (Social Networking Service) and the Web, including 98.2% words (9,117 / 9,287) used in this topic modeling.

Figure 5 shows the results of 1st layer NMF, which indicate the optimal numbers of window topics are ranging from 10 to 25 sporadically as previous research [3]. Figure 6 shows the results of 2nd layer NMF by changing *k′* ∈ [25, 90] and *m′* ∈ {10,15,20}, and we concluded the score of *k′* = 29 is the highest consistently for both *m′* = 10 (0.22) and *m′* = 15 (0.19) and use *m′* = 10 in the following analysis.

As a supplement, decreasing trends of Figure 6 are unlike previous study, so we conducted *k′* ∈ [5, 24]. While this resulted in *k′* = 10 as high score, we decided not to use because we can’t see the notable increasing or decreasing trend for each topic, and it’s difficult to interpret such a little topic which are somewhat abstract. Although these scores (score = 0.217) and number of dynamic topics (*k′* = 29) are a bit lower than previous study (*k′* = 57, *score* = 0.364), we think that narrowing down to specific theme “older drivers” affects *k′*, and using whole Japanese SNS corpus, not specific to older drivers, affects the lower coherence score.


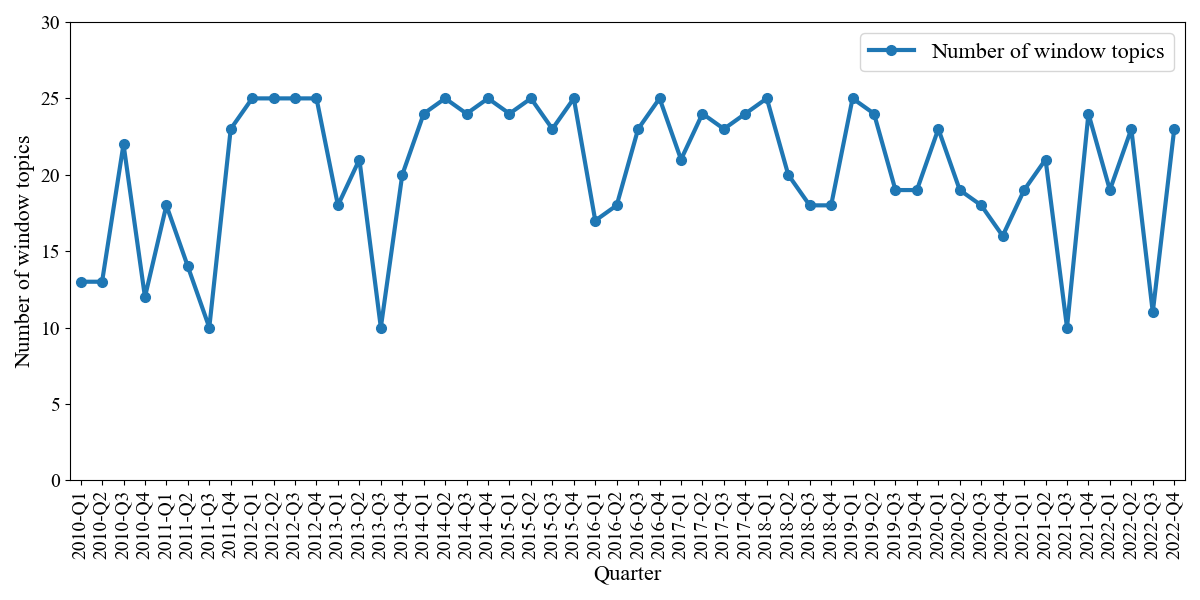


Figure S5: Number of window topics identified from 2010 to 2022


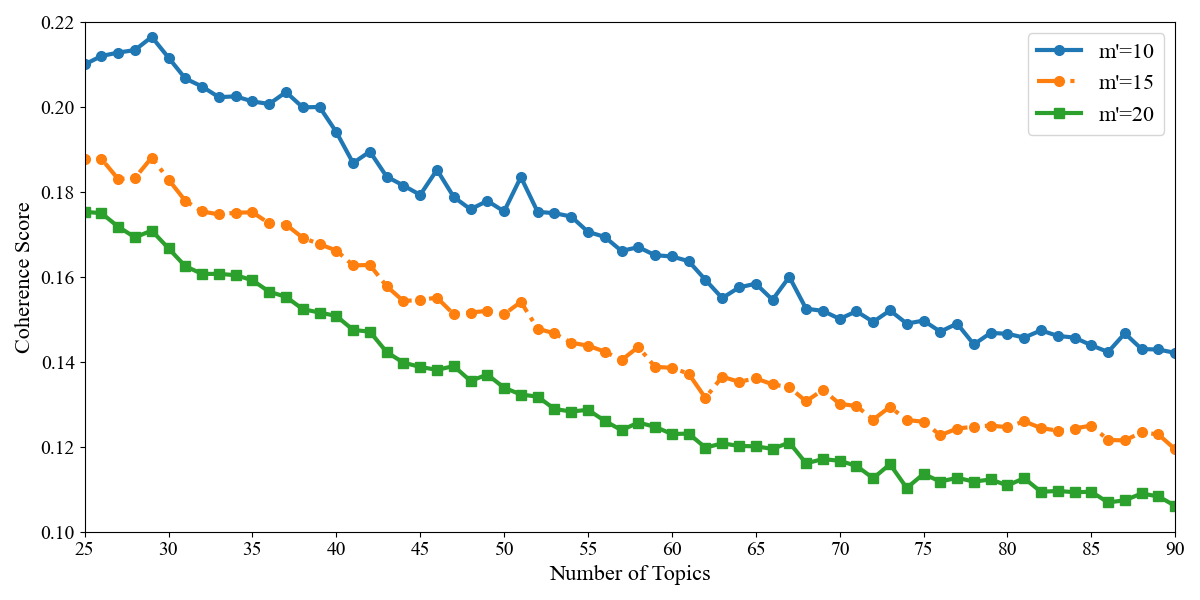


Figure S6: Number of dynamic topics ranging [25,90] in *m′* = {10, 15, 20}

### S3.2 Results of Two Layers of NMF

Table S3 shows the all topic descriptions with all 29 topics. Table S4 shows example tweets of selected 16 topics, and those topics not mentioned in the main text are shown in Table S5 and Figure S7. Figure S7 presents the temporal trends of topic proportions for 7 topics, where the R-squared value is under 0.1.

Table S3: Top 10 weighted words of all topics with the mean and standard deviation (N=614,429).

| Topic | Top 10 weighted words in Japanese | Mean  (%) | SD  (%) |
| --- | --- | --- | --- |
| **1** | 返納 免許 自主 取り上げる 制度 強制 返上 証明 剥奪 割引 | 7.0 | 14.2 |
| **2** | 事故 起こす 増える 交通 報道 死亡 減る 多発 起きる 防止 | 5.8 | 10.2 |
| **3** | 思う 良い 自分 必要 死ぬ 年齢 若い 出来るなー 考える | 5.3 | 9.7 |
| **4** | 多い 最近 田舎 特に 悪い マナー 若い 過ぎる 結構 若者 | 4.4 | 10.2 |
| **5** | 交通 安全 運動 防止 自転車 飲酒 全国 歩行 公共 子ども | 4.1 | 7.9 |
| **6** | 暴走 池袋 東京 飯塚 nhk 被告 事件 遺族 幸三 慰霊 | 4.0 | 9.2 |
| **7** | 自動 社会日本 技術 必要 安全 早い 実験 普及 開発 | 3.9 | 8.9 |
| **8** | 問題 社会 考える 生活 田舎 解決 必要 難しい 交通 地方 | 3.9 | 8.3 |
| **9** | 更新 免許 講習 受ける 試験 検査 センター 優良 天皇陛下 神戸 | 3.8 | 9.1 |
| **10** | 言う 悪い 聞く 無い 行く 自分 来る 同じ 出来る 若い | 3.8 | 8.5 |
| **11** | ニュース yahoo nhk 産経新聞 tbs デジタル 朝日新聞 jnn 産経 news | 3.8 | 8.1 |
| **12** | 走る 道路 高速 車線 衝突 キロ 正面 後ろ 信号 対向 | 3.6 | 8.2 |
| **13** | 見る 出る 周り 駐車 ニュース 来る 初めて 信号 後ろ 見える | 3.6 | 7.9 |
| **14** | ブレーキ アクセル 踏む 間違える 間違い ペダル 突っ込む 駐車 mt 操作 | 3.5 | 10.4 |
| **15** | 自分 大丈夫 自信 悪い 考える 欲しい 出来る 良い 家族 マナー | 3.5 | 6.8 |
| **16** | 怖い マジ 本当に 自転車 田舎 ホント 一番 最近 轢く 駐車 | 3.5 | 11.1 |
| **17** | タクシー 聞く会社 進む コロナ 行く 感染 使う女性 新聞 (taxi, ask company, move forward, corona, go, infection, use woman, newspaper) | 3.1 | 7.2 |
| **18** | 突っ込む 男性 コンビニ プリウス 病院 スーパー 駐車 女性 停止 アクア (crash, man, convenience store, prius, hospital, supermarket, parking, woman, stop, aqua) | 3.1 | 7.4 |
| **19** | 歩道 横断 信号 渡る 無視 自転車 歩行 轢く 止まる 交差点 (sidewalk, cross, signal, crosswalk, ignore, bicycle, pedestrian, run over, stop, intersection) | 3.0 | 6.8 |
| **20** | バス 行く 田舎 乗る 送迎 時間 路線 乗客 座る 公共 (bus, go, countryside, ride, shuttle, time, route, passenger, sit, public) | 3.0 | 6.8 |
| **21** | 危険 自転車 若者 信号 プリウス オスプレイ 感じる 判断 パニック家族 (danger, bicycle, young people, signal, prius, osprey, feel, judge, panic, family) | 2.9 | 7.9 |
| **22** | 認知 検査 機能 恐れ 疑い 警察庁 強化 改正 技能 半数 (cognition, test, function, fear, suspicion, national police agency, strengthen, revision, skill, half) | 2.9 | 7.4 |
| **23** | 死亡 女性 男性 衝突 逮捕 乗用車 バイク トラック ひき逃げ 正面 (death, woman, man, collision, arrest, passenger car, motorcycle, truck, hit-and-run, frontal) | 2.7 | 6.4 |
| **24** | マーク 付ける 初心者 貼る 標識 新しい 枯葉 四つ 若葉 後ろ (mark, attach, beginner, stick, sign, new, withered leaf, four, fresh leaf, back) | 2.4 | 8.5 |
| **25** | 乗る プリウス 自転車 バス mt 悪い 欲しい タクシー 子供 マルチリンガル (ride, prius, bicycle, bus, manual transmission, bad, want, taxi, child, multilingual) | 2.4 | 5.8 |
| **26** | 自動車 増える 保険 値上げ 社会 増加 若者 考える 大手 対象 (automobile, increase, insurance, price increase, society, growth, young people, consider, major companies, target) | 2.3 | 4.8 |
| **27** | 今日 行く 来る ホーム 講習 出る 仕事 時間 車線 最近 (today, go, come, home, training, leave, work, time, lane, recently) | 2.0 | 3.7 |
| **28** | 危ない 本当に 自転車 マジ 欲しい 信号 実態 出る 最近 子供 (dangerous, really, bicycle, seriously, want, signal, reality, appear, recently, child) | 1.8 | 4.5 |
| **29** | 知る 電話 ラジオ 狂う 心配 ハイウェイ 掛ける 携帯 号線 困る (know, phone, radio, go crazy, worry, highway, call, mobile, route, trouble) | 0.7 | 3.5 |

Table S4. Top 16 topics with highly weighted example tweets.

| No. | Topic name | Representative tweet | Representative tweet (English) |
| --- | --- | --- | --- |
| 1 | *License surrender* | 高齢ドライバーの運転免許自主返納、どれくらいの人がしてるの | How many older drivers voluntarily surrender their licenses? |
| 2 | *Crash events* | 高齢者ドライバーの事故覚悟で特攻してくるのほんとやめてほしい | I really wish older drivers would stop driving recklessly as if prepared for an accident. |
| 3 | *Thoughts on older drivers* | 多分運転してたのは老人の人だと思う | I think the driver was probably an older adult. |
| 4 | *Prevalent older drivers* | それはひどい 老人ドライバーは気が短い人が多い気がする。 | That's terrible. I feel like many older drivers are short-tempered. |
| 5 | *Traffic safety* | 秋の交通安全運動 子どもと高齢者守れ 脇見運転防止に重点 大分合同新聞 | Autumn traffic safety campaign: Protect children and the elderly, focus on preventing distracted driving. Oita Godo Shimbun |
| 6 | *Ikebukuro incident* | 悲報81歳の老人が運転する車が暴走、うん千万円のビンテージカーに着地して廃車にする | Breaking news: An 81-year-old driver lost control of their car and crashed into a vintage car worth tens of millions, rendering it a total loss. |
| 7 | *Self-driving technology* | 高齢者の足代わりに日立、自動運転ロボカー:日本経済新聞 | Hitachi introduces a self-driving robot-car as a mobility aid for elderly people. (Nikkei) |
| 8 | *Social issues* | そうですか 運転手は80代の方のようなのでまた高齢者の運転問題で騒がれそうですね。 | I see, the driver appears to be in their 80s, so the issue of older drivers will likely be debated again. |
| 9 | *License renewal* | 免許の更新。神戸優良高齢運転者運転免許更新センター | License renewal at the Kobe Older drivers' License Renewal Center. |
| 10 | *Discussing senior driving* | 高齢者運転……ウチのじぃちゃんにも言うた方がええんかなぁ | Senior driving… Maybe I should talk to my grandpa about it too. |
| 11 | *News media* | 94歳が運転90歳はね死なす 2015年11月28日土 yahooニュース 老人のバトルロワイヤルが始まった | A 94-year-old driver hit and killed a 90-year-old. (Yahoo News, Nov 28, 2015) The elderly battle royale has begun. |
| 12 | *Road traffic crash* | 70代の高齢ドライバー、高速道路の追い越し車線をヘッドライトをつけて逆走 | A driver in their 70s was driving the wrong way in the passing lane on the highway with headlights on. |
| 13 | *Witnessing older drivers* | 高齢者が運転されている車を見ますとぶつけたり擦れたりしている車がそうした例に繋がりますね | When I see cars driven by elderly people, they often have dents or scratches, which makes sense. |
| 14 | *Driving errors* | なぜ高齢者はアクセルとブレーキをこんなに踏み間違えるんだ今まで何十年も運転してきて癖みたいになってるはずなのに | Why do elderly drivers mix up the accelerator and brake so often? After decades of driving, it should be second nature. |
| 15 | *Self-confidence* | 老人かやってたわ老人の運転のなにがやばいって自分が老人になってるよってことに気付いてない | The problem with older drivers is that they don’t realize they have become elderly themselves. |
| 16 | *Fear of crashes* | 高齢者の運転の方が違法改造より怖い | Senior driving is scarier than illegally modified cars. |

Table S5. Description of nine topics, including topic names, top 10 weighted words, proportions, and results of linear regression (the formula $Y_{t}= \beta_{0}+\beta_{1}X_{t}+\epsilon_{t}$).

| Topic name | Top 10 weighted words | Proportion | $\beta_{0}$ | $\beta_{1}$ | R^2^ | *P* value |
| --- | --- | --- | --- | --- | --- | --- |
| Topic 3:  *Thoughts on older drivers* | think good oneself necessary die age young can hmm consider | 5.26 | 5.54 | -0.01 | 0.009 | 0.505 |
| Topic 4:  *Discussing senior driving* | many recently rural especially bad manners young too(much) quite youth | 4.38 | 4.65 | -0.01 | 0.016 | 0.375 |
| Topic 11:  *News media* | news Yahoo NHK Sankei-newspaper Asahi digital Asahi newspaper JNN Sankei-news | 3.78 | 3.98 | -0.02 | 0.034 | 0.189 |
| Topic 12:  *Road traffic crash* | run road highway lane collision km head-on behind light opposite | 3.64 | 3.33 | 0.03 | 0.07 | 0.058 |
| Topic 13:  *Witnessing older drivers* | see left around parking news come first light behind visible | 3.62 | 4.12 | -0.01 | 0.015 | 0.38 |
| Topic 15:  *Self-confidence* | oneself OK confidence bad consider want can good family manners | 3.52 | 2.18 | 0.03 | 0.094 | 0.027 |
| Topic 16:  *Fear of crashes* | fear seriously really bicycle rural really the-most recently hit parking | 3.51 | 3.65 | 0.00 | 0.000 | 0.978 |


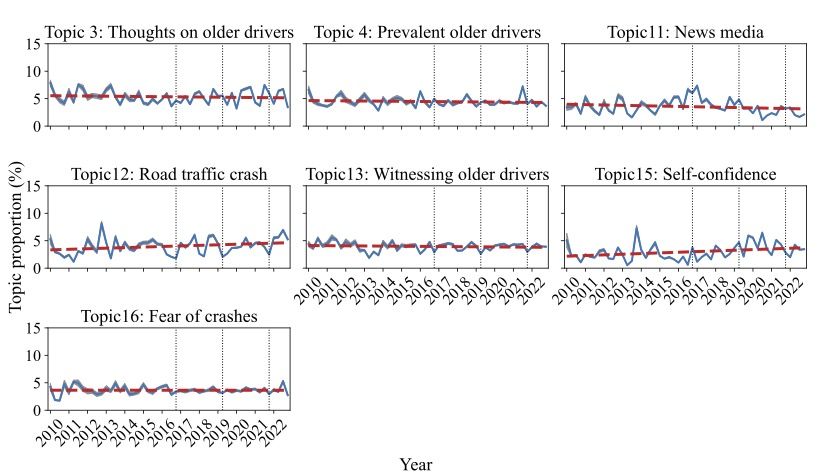


Figure S7: Temporal trends of seven topic mean proportions, shown as solid blue lines, with a red dashed line representing the regression line. Gray shaded areas represent 95% confidence intervals computed using standard error of the mean.

# References

1. SourceForge.jp. Japanese stopword list, 2024. URL: [hhttp://svn.sourceforge.jp/svnroot/slothlib/CSharp/Version1/SlothLib/NLP/Filter/StopWord/word/Japanese.txt](https://github.com/neologd/mecab-unidic-neologd) [accessed 2024-07-10].
2. Matsuo A, Sasahara K, Taguchi Y, Karasawa M. Development and validation of the Japanese moral foundations dictionary. PLoS ONE 2019; 14(3):e0213343 [doi: 10.1371/journal.pone.0213343]
3. Greene D, Cross JP. Exploring the political agenda of the European parliament using a dynamic topic modeling approach. Political Analysis 2017;25(1):77–94 [doi: 10.1017/pan.2016.7]
4. Lee D, Seung H. Learning the parts of objects by non-negative matrix factorization. Nature 1999;401:788–791 [doi: 10.1038/44565]
5. O’Callaghan D, Greene D, Carthy J, Cunningham P. An analysis of the coherence of descriptors in topic modeling.

Expert Syst. Appl 2015;42(13):5645–5657 [doi: 10.1016/j.eswa.2015.02.055]

1. Sakaki T, Mizuki S, Gunji N. BERT pre-trained model trained on large-scale Japanese social media corpus, 2019. URL: https://github.com/hottolink/hottoSNS-bert [accessed: 2024-07-18]
